# Global Bias-Corrected CORDEX Datasets at Half Degree Resolution

**DOI:** 10.1038/s41597-025-06200-4

**Published:** 2025-11-12

**Authors:** Fuseini Yakubu, Jürgen Böhner, Udo Schickhoff, Thomas Scholten, Shabeh ul Hasson

**Affiliations:** 1 HAREME Lab, Institute of Geography, ESRAH, Universität Hamburg, Hamburg, Germany; 2 Institute of Geography, ESRAH, Universität Hamburg, Hamburg, Germany; 3 Department of Geosciences, Chair of Soil Science and Geomorphology, University of Tübingen, Tübingen, Germany

**Keywords:** Climate sciences, Projection and prediction

## Abstract

Although the Coordinated Regional Climate Downscaling Experiments (CORDEX) consistently provide dynamically downscaled high-resolution climate data, their biases are nonetheless corrected against subjectively selected observational datasets and varying bias-correction methods, making impact assessments inconsistent. Impact assessment communities, such as the Intersectoral Impact Model Intercomparison Project (ISIMIP), prefer consistent climate simulations that are corrected for biases using the same method against a common set of observational datasets. We present the quasi-global datasets of GloBCORD-HD, prepared at a daily temporal and 0.5° spatial resolution for the historical period (1950–2019) and the future period (2020–2099) for three Representative Concentration Pathways (RCPs) 2.6, 4.5, and 8.5. Three datasets were developed by bias-correcting CORDEX experiments, which dynamically downscaled the Earth System Models of MPI-M-MPI-ESM-LR, ICHEC-EC-EARTH, and NOAA-GFDL-GFDL-ESM2M over the maximum number of regional domains, against the single observational dataset GSWP3-W5E5 v2.0, using the same ISIMIP3BASD v2.5 bias correction algorithm. Comprehensive validation demonstrates that GloBCORD-HD reduces biases, improves regional extremes representation, enhances climate signals consistency, with seamless boundary transitions, and enables robust global impact assessments.

## Background & Summary

Impact assessment provides insights into the potential socioeconomic and environmental consequences of adverse climatic changes and feedback of adaptive measures, and thus, is crucial for informed decision-making^1^. However, robust impact assessment requires high-resolution climate and climatic change information at local scales. Although Earth System Models (ESMs) inform on large-scale climatic changes, they usually lack the representative spatiotemporal accuracy and details needed for robust impact assessments^2^. Conversely, Regional Climate Models (RCMs) better capture topoclimatic heterogeneity unresolved by ESMs, including complex topography, coastlines, land use patterns, and small-scale atmospheric phenomena^3–5^, thus improving simulation of mean and extreme hydroclimatic processes at regional to kilometre scales while maintaining consistency with broader ESM patterns^3,6–10^. Studies suggest that higher spatial resolution in RCMs improves the representation of extreme weather events, such as heatwaves, heavy precipitation, and storms^11–13^. This emphasizes that high-resolution climate data is crucial for robust localized impact assessments^14^.

To produce high-resolution climate and climatic change projections, RCMs are used to downscale the ESMs experiments over certain regions either dynamically or statistically. Such spatial refinements depend on inconsistent choices of ESM, RCM, and most importantly, the downscaling framework, such as spatiotemporal resolutions, domain extents, and model physics suite. Individual efforts further lack best practices for producing and disseminating the data, hindering its accessibility to a wider impact assessment community. Against this background, the Coordinated Regional Climate Downscaling Experiment (CORDEX)^12^ mandated an internationally coordinated effort to produce high-resolution climate change projections for about 14 regional domains with quasi-global coverage from objectively selected ESMs using a common downscaling framework for input into impact and adaptation studies^12,15–17^. The first phase of CORDEX^12^ downscaled a subset of ESMs participating in the Coupled Model Intercomparison Project Phase 5 (CMIP5)^18^ at 0.44° resolution for historical and future climates under different representative concentration pathways (RCPs)^19^. The number of simulations and driving ESMs in the first phase of CORDEX-CMIP5 is different across the regional domains, making consistent impact assessment across the globe difficult^20^. The CORDEX^12^ experiments have been very helpful, particularly in regions with complex topography and heterogeneous land surfaces, where fine-scale processes significantly shape local climates^4^. For example, Fotso-Nguemo *et al*. (2017) demonstrated that CORDEX experiments with REMO RCM captured more detailed spatial variations for regions with complex topography over Central Africa than ESMs^21^. Teichmann *et al*. (2020) also showed that the CORDEX^12^ ensembles generally represent well the spread of temperature and precipitation changes from the CMIP5^18^ ESMs. Besides finer spatial resolutions of 0.44° and 0.22° with spatially and physically coherent outputs^6^, the CORDEX^12^ experiments still exhibit biases against observed mean and extreme climatic changes, particularly over complex topography that can substantially distort climate impact assessments^4,22,23^. For instance, Hasson *et al*. (2019) showed that CORDEX-CMIP5 experiments over the South Asia domain featured larger cold and wet biases than their driving ESMs. These biases are either inherited from their driving ESMs that are not supposed to be corrected by RCMs, or stem from the RCM structure itself, or a combination of both^24^. Adjusting these biases, however, is essential for their use in the climate impact studies^25–28^.

Statistical bias adjustment that ranges from adjusting simple means to trend-preserving multivariate quantile mapping has become a common post-processing technique to enhance the utility of climate model outputs for impact studies^14,24,29,30^. It aligns simulated historical climates with observations and improves the accuracy of future climate projections^26,30,31^. For example, Rajczak and Schär (2017) demonstrated that bias-corrected CORDEX^12^ datasets provided superior simulations of extreme precipitation events in Europe compared to coarser models. Navarro-Racines *et al*. (2020), Xu *et al*. (2021), and Chen *et al*. (2024) have all demonstrated that bias correction led to more accurate and realistic future climate projections^26,32,33^. Despite such improvements in the statistical representation of climate data, bias adjustment does not correct all fundamental model errors and can, in some cases, modify future climate projections^34,35^. Furthermore, the skill of each bias correction method differs, featuring certain advantages or disadvantages over the other, and should be applied with a clear understanding of its limitations, nature of the target variable, and the underlying climate models^36^.

Although the CORDEX^12^ framework provides consistent dynamically downscaled high-resolution climate data, individual impact assessment studies nevertheless correct these biases against subjectively selected observational datasets and varying bias-correction methods, making impact assessments inconsistent. In contrast, impact assessment communities, such as the Intersectoral Impact Model Intercomparison Project (ISIMIP), prefer consistent climate simulations that are corrected for biases using a uniform robust method against a common set of observational datasets^37–39^. Besides, CORDEX-CMIP5 is dynamically downscaled at 0.44° resolution, but a comprehensive and globally consistent bias-corrected dataset encompassing all key variables remains lacking. This gap underscores the need for a standardized, high-resolution dataset for robust regional and global climate change assessments across the globe^37,38^.

Here, we present the quasi-global datasets of GloBCORD-HD^40^, prepared at a daily temporal and 0.5° spatial resolution for the historical period 1950–2019, and the future period 2020–2099 for three Representative Concentration Pathways (RCPs) 2.6, 4.5, and 8.5. The datasets were developed by bias-correcting CORDEX^12^ experiments, which dynamically downscaled the MPI-M-MPI-ESM-LR^39^, ICHEC-EC-EARTH^41,42^, and NOAA-GFDL-GFDL-ESM2M^43,44^ ESMs over the maximum number of regional domains, whether by a single or multiple RCMs, against a single observational dataset, GSWP3-W5E5 v2.0^45,46^, using the same ISIMIP3BASD v2.5^47,48^ bias-correction algorithm for up to seven key variables for impact assessment. The advanced algorithm of ISIMIP3BASD v2.5 ensures robust bias correction using a variable-specific trend-preserving quantile mapping approach, making the GloBCORD-HD^40^ dataset particularly valuable for future climate projections. These GloBCORD-HD^40^ datasets significantly reduce biases, boundary discontinuities, and decrease overall data volume by removing CORDEX^12^ domain overlaps, and provides consistent input data for robust climate impact assessments. Unlike statistically downscaled ESMs datasets, such as ISIMIP3b^49^ and NEX-GDDP (https://registry.opendata.aws/nex-gddp-cmip6, last access: 12 February 2025), GloBCORD-HD^40^ does not aim to provide higher-resolution datasets but instead focuses on ensuring the robust use of existing dynamically downscaled CORDEX^12^ datasets, particularly for impact studies. Consequently, it complements rather than competes with existing datasets at the same resolution, in facilitating evidence-based adaptation planning and policy decisions across all sectors, particularly for water resources, agriculture, energy, health, and ecosystems^5,50–53^.

## Methods

### Observational climate dataset

We utilized the existing GSWP3-W5E5 v2.0^45,46^ observational dataset from the ISIMIP repository (10.48364/ISIMIP.982724.2), which is available at a daily temporal and 0.5° spatial resolution covering the period from 1901 to 2019. The GSWP3-W5E5 v2.0^45,46^ dataset was selected for bias correction because it has been used as a primary input for impact model simulations under the ISIMIP3a protocol^54^ and provides coverage for several impact assessment variables. The dataset also serves as the standard baseline climate dataset for the Open Global Glacier Model (OGGM)^55^. The GSWP3-W5E5 v2.0^45,46^ dataset is a merger of two datasets, namely, the GSWP3 v1.09^56^ and W5E5 v2.0 datasets^45,46^.

The GSWP3 v1.09^56^ is the bias-adjusted version of the dynamically downscaled Twentieth Century Reanalysis version 2^57^, available at 3-hourly temporal and 0.5° spatial resolution across the globe for the period 1901–2010. Whereas the W5E5 v2.0^45,46^ is the daily global dataset at 0.5° spatial resolution spanning over 1979–2019 period. It was prepared by applying the WATCH Forcing Data methodology (WFDEI^58^; to the fifth-generation European Reanalysis dataset (ERA5)^59^ and the precipitation data of the Global Precipitation Climatology Project (GPCP) version 2.3^60^). The GSWP3-W5E5 v2.0^45,46^ combines the W5E5 v2.0^45,46^ dataset for 1979–2019 with the GSWP3 v1.09^56^ dataset for 1901–1978. The latter was bias-adjusted towards the W5E5 v2.0^45,46^ for homogenization using ISIMIP3BASD v2.5^47,48^.

We selected the daily mean near-surface air temperature (tas), daily minimum near-surface air temperature (tasmin), daily maximum near-surface air temperature (tasmax), daily total precipitation (pr), daily mean surface downwelling longwave radiation (rlds), daily mean near-surface relative humidity (hurs), and daily mean near-surface surface winds (sfcWind) from the GSWP3-W5E5 v2.0^45,46^ observational dataset.

### Dynamically downscaled CORDEX experiments

The ESM-RCM pairs were selected based on three key criteria to ensure comprehensive spatial and temporal coverage. First, we included only those experiments that downscaled a particular ESM for at least five regional domains, regardless of whether achieved by a single RCM or multiple RCMs. Second, datasets must provide complete daily historical simulations and at least two future scenario projections (RCP2.6, RCP4.5, or RCP8.5) for essential climate variables, including mean, maximum, and minimum temperature, precipitation, snow flux, specific and relative humidity, downwelling shortwave radiation, upwelling longwave radiation, surface pressure, and surface wind speed. Third, datasets must contain data for at least five of these specified variables across the study domains, with a minimum of one realization, and preference given to multi-realization ensembles. Based on the criteria, three dynamically downscaled ESM experiments, performed under the CORDEX^12^ framework using five RCMs, were chosen. These dynamically downscaled ESMs include the MPI-M-MPI-ESM-LR^39^, ICHEC-EC-EARTH^41,42^, and NOAA-GFDL-GFDL-ESM2M^43,44^. In addition to scenario uncertainty, the selected ESMs also covered a wide range of climate sensitivity. For instance, the Equilibrium Climate Sensitivity (ECS) of NOAA-GFDL-GFDL-ESM2M^43^,^44^, ICHEC-EC-EARTH^41^,^42^,and MPI-M-MPI-ESM-LR^39^ is ~2.4K, ~3.2K, and ~3.6K, respectively^61^–^63^. This range encompasses low to moderate-high ECS values of CMIP5 models, ensuring that the bias-corrected dataset accurately represents the diverse responses of models to greenhouse gas forcing and provides users with a robust sampling of structural model uncertainty. We herein refer to CORDEX^12^ versions of the dynamically downscaled ESMs that form GloBCORD-HD^40^ as MPI-LR_CDX, EC-EARTH_CDX, and GFDL_CDX.

**Fig. 1:**
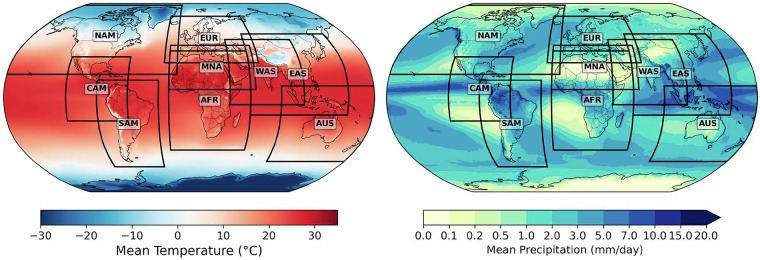
Spatial distribution of mean temperature (°C, left) and mean precipitation (mmd^−1^, right) from the GSWP3-W5E5 v2.0 observational dataset over 1960-2019. Black rectangles indicate the CORDEX domains used for regional climate model simulations, that includes North America (NAM), Central America (CAM), South America (SAM), Europe (EUR), Africa (AFR), Middle East and North Africa (MNA), East Asia (EAS), West Asia (WAS), and Australasia (AUS).

Their simulation length typically spans from 1951 to 2005 for the historical period and from 2006 to 2100 for three future climate change scenarios: RCP2.6, RCP4.5, and RCP8.5. An exception to this is the historical period of the West Asia domain in MPI-LR_CDX, which starts only from 1961. Therefore, the historical period for the MPI-LR_CDX for all domains has been restricted to the 1961–2005 period. The RCP2.6 scenario of the GFDL_CDX was not available. For each considered ESM, we downloaded their CORDEX^12^ downscaled simulations for the maximum number of regional domains to provide a quasi-global coverage for our final dataset, excluding the polar regions (Fig. 1). For the selected regions, CORDEX^12^ dynamically downscaled the ICHEC-EC-EARTH^41,42^ over nine regional domains, and they include Africa (AFR), Australasia (AUS), Central America (CAM), East Asia (EAS), Europe (EUR), Middle East North Africa (MNA), North America (NAM), South America (SAM), and West Asia (WAS). However, the MPI-LR_CDX had eight out of these nine domains, MNA was not available, and the GFDL_CDX had five to seven out of these nine domains. EAS and AUS domains  were not available entirely, including NAM and CAM domains for the RCP4.5 scenario for the GFDL_CDX. It is pertinent to mention that the EUR, WAS, and AFR domains completely overlap with the MNA domain. Hence, MPI-LR_CDX coverage remains the same as that of EC-EARTH_CDX, even though the MNA domain was not available. We downloaded the same set of daily variables as the observational dataset, such as tas, tasmax, tasmin, pr, hurs, rlds, and sfcWind for the full period of record from the CORDEX^12^ archive. The latter two variables were not available for the GFDL_CDX in the CORDEX^12^ archives. The variable hurs was not also available over EAS and AUS for the EC-EARTH_CDX and MPI-LR_CDX in the CORDEX^12^ archives. The individual RCMs making the GloBCORD-HD^40^ are: RCA4^64^, REMO2009^65^, CCLM4–8–17-CLM3-5^66^, CRCM5^67^, WRF (https://www2.mmm.ucar.edu/wrf/users/download/get_sources.html, last access: 12 February 2025) and CCLM5-0-2 (http://www.clm-community.eu/, last access: 12 February 2025). The raw CORDEX^12^ experiments were obtained from the metagrid portal of the Earth System Grid Federation (ESGF) and can be accessed at (https://esg-dn1.nsc.liu.se/search/cordex/).

These seven variables in GloBCORD-HD^40^ were selected based on their critical importance for climate impact assessments across multiple sectors, including agriculture, hydrology, energy, and human health. They represent some of the most frequently requested parameters by the impact modelling community. While we initially planned to include up to ten variables by adding rsds (surface downwelling shortwave radiation), prsn (snowfall flux), and ps (surface pressure), computational, storage, and time constraints within the project timeline necessitated prioritizing the current variables. Figure 1 shows the temperature and precipitation climatologies from the GSWP3-W5E5 v2.0^45,46^ for the 1960–2019 period and the CORDEX^12^ domains used in producing the dataset. The ESMs and their downscaled RCMs, constituting the final datasets, are also summarized in Table 1.

**Table 1:** List of three GloBCORD-HD datasets, which are prepared from the three dynamically downscaled Earth System Models by each Regional Climate Model over distinct domains, yielding a quasi-global coverage.

No	Downscaled ESM and Scenarios	Run	Downscaling RCM/Version	CORDEX Domains									Variables
				AFR	AUS	CAM	EAS	EUR	MNA	NAM	SAM	WAS	
1	MPI-M-MPI-ESM-LR (Historical, RCP2.6, RCP4.5, RCP8.5)	r1i1p1	REMO2009, v1	X								X	tas, tasmax, tasmin, pr, hurs, rlds, sfcWind
			RCA4, v1			X		X			X		
			CCLM4-8-17-CLM3-5, v1		X								
			CCLM5-0-2, v1				X						
			CRCM5, v1							X			
2	ICHEC-EC-EARTH (Historical, RCP2.6, RCP4.5, RCP8.5)	r12i1p1	RCA4, v1	X		X		X	X	X	X	X	
			CCLM4-8-17-CLM3-5, v1		X								
			CCLM5-0-2, v1				X						
3	NOAA-GFDL-GFDL-ESM2M (Historical, RCP4.5, RCP8.5)	r1i1p1	RCA4, v1	X		X		X	X	X^+^	X	X	tas, pr, tasmax, tasmin, hurs
			WRF, v3-5-1							X^*^			

The historical period includes 70 years for EC-EARTH_CDX, GFDL_CDX, and 60 years for MPI-LR_CDX and 80 years for the future period for all. Note: GFDL_CDX pr downscaled with RCA4^+^ was not available over NAM, which is taken from GFDL_CDX downscaled with WRF^*^, hurs was not available over EAS and AUS domains.

### Preprocessing dataset

Following Leal Filho *et al*. (2024), we extended the historical simulations for all three ESMs to 2019 by appending data from the RCP8.5 scenario (2006–2019)^68^. We used the RCP8.5 scenario because it was the most consistent for all variables among all ESM-RCM combinations and RCP scenarios. The RCP8.5 also showed a minimal divergence in the first two decades of the 21st century due to similar near-term greenhouse gas concentrations and climate system inertia^69–72^. This period also overlaps with the most recent observational record, maximizing the potential benefits. The year 1961 was duplicated as 1960 for the WAS domain, and 1951 for 1950 for all other experiments to complete the historical period to 60 years for MPI-LR_CDX and 70 years for other ESM experiments. The RCP datasets then spanned 80 years, starting from 2020 and ending in 2099.

All gaps in the daily CORDEX^12^ data were filled by duplicating data from the preceding day. For instance, the GFDL_CDX dataset features a 365-day calendar and does not have leap days. We added leap days to GFDL_CDX by duplicating February 28 to February 29. This extended the historical simulations by 17 days and the RCP scenarios (RCP8.5 and RCP4.5) by 20 days per variable. This ensured consistency with the observational data, avoided processing errors during bias correction, and allowed direct comparison of all datasets. All the datasets were regridded from their native resolution to the resolution of the observational dataset at a regular 0.5° grid using the Climate Data Operators (CDO) version 2.4.1^73^. We used conservative remapping for *pr* and *rlds* to preserve their quantities^74^ and bilinear remapping for the other continuous variables to preserve gradients without introducing artificial discontinuities^75^.

### Bias correction methodology

We employed the Inter-Sectoral Impact Model Intercomparison Project **(**ISIMIP) bias correction methodology because the community has significantly advanced the development and refinement of bias adjustment methods over time^26^, evident from the ISIMIP Fast Track^76^, to subsequent updates in the ISIMIP2^77^ and ISIMIP3 protocols^48^. These updates ensure the bias adjustment methods remain current and incorporate the latest advancements in the field^26^. We used bias adjustment and statistical downscaling algorithm version 2.5 (ISIMIP3BASD v2.5^47^), which was recently employed in the round of impact model simulations under the ISIMIP3a protocol. The algorithm is publicly available at 10.5281/zenodo.4686991. The ibicus Python package used in this bias correction can be accessed at (https://github.com/ecmwf-projects/ibicus, last access: 12 February 2025).

By incorporating physical considerations and maintaining an appropriate level of complexity, the ISIMIP3BASD^48^ algorithm provides a well-balanced approach that effectively reduces bias while ensuring future projections are adjusted to reflect more accurately expected future conditions. The method is a parametric quantile mapping designed to robustly adjust biases in all percentiles of a distribution and preserve trends in these percentiles^48^. Unlike rivals, ISIMIP3’s quantile mapping approach provides a more detailed and nuanced correction by considering every quantile individually^48^. It also allows for different fitting options, such as parametric or non-parametric fits, to ensure the best possible representation of the observed data^26,29,48^. This flexibility enables the method to adapt to different variable characteristics and improve the accuracy of the bias-adjusted simulations. This enables more robust bias adjustment of extreme values^26,48^. Further, the algorithm ensures that the trend existing in the raw simulations is preserved during the bias correction process. This is crucial for capturing the long-term changes in climate variables and their impacts on various sectors^26,48^. For precipitation, a threshold of 0.1 mm/day is used to correct the issue of drizzle commonly observed in simulations, where very low precipitation amounts are overestimated^48^. Furthermore, correcting tasmax and tasmin independently can potentially lead to inconsistencies, such as tasmax being less than tasmin or generating unrealistic diurnal temperature ranges. Therefore, the ISIMIP3BASD^48^ instead employs an indirect approach to bias correct tasmax and tasmin by correcting mean temperature, diurnal temperature range, and temperature skewness (tas, tasrange, tasskew). The tasrange is calculated as tasmax - tasmin, and tasskew as ((tas - tasmin) / tasrange). After correcting tas, tasrange, and tasskew, the same formulas are used to recalculate tasmax and tasmin, which are bias-corrected and preserve their relationship.

The method’s multivariate bias adjustment component, while designed to maintain inter-variable physical consistency, has demonstrated practical issues including spatial incoherence and overfitting artifacts, forcing many users to rely solely on univariate corrections^78^. Despite its trend-preserving design, the univariate ISIMIP3BASD^48^ algorithm can exhibit several significant limitations that affect its robustness and applicability. Empirical evaluations in Spruler *et al*. 2023 reveal that the method can modify climate change trends by up to 50%, particularly for threshold-sensitive indices such as dry days, undermining its core trend-preservation principle^29^. The algorithm’s reliance on parametric distributions (normal or gamma) may inadequately represent extreme values and tail behaviours, leading to poor performance in correcting climate extremes^29^. These limitations highlight the need for rigorous, use-case-specific evaluation and caution against the wholesale application of the method across all climate applications^29^.

Compared to other alternative bias correction methods, such as quantile delta mapping, quantile mapping, and linear scaling, the ISIMIP3BASD v2.5^47^ demonstrated superior performance in reducing the risk of artificial trend distortions and in preserving trends during tests with samples of our input data. The impact assessment communities widely use this method due to its trend-preserving properties and suitability for climate change studies^13,29^. We considered the full length of the historical period (60–70 years) as the training period for bias correction and the full length of the future period (80 years) for the application period using the univariate bias correction method.

### Postprocessing datasets

The consistency of all variables was verified to ensure they fall within the valid ranges as mentioned in Lange *et al*. (2021). Only a minimal number of values for pr, sfcWind, tasmax, and tasmin exceeded the variable ranges set by ISIMIP3BASD^48^, and these outliers were clipped to the allowed limits, with minimal impact on the overall dataset. The individual bias-corrected regional domains were then merged into a global grid using the ensemble mean approach in CDO^73^. Since the observational data used had no discontinuities, the corrected historical datasets were very close to the observations, and the ensemble mean generated smooth datasets over the overlapping regions of CORDEX^12^ domains (Supplementary Fig. 1). However, different future change signals simulated by individual RCMs, although smoothed out over the overlapping areas using ensemble means, may still introduce some discontinuities along the boundaries of overlapping areas (Supplementary Figs. 1 and 2), which are predominantly located over the oceanic regions (e.g., the eastern and western boundaries of CAM, NAM, SAM, AFR, EUR, EAS, and AUS domains, and southern WAS and MNA and southern and northern boundaries of EAS). Since GloBCORD-HD^40^ is a land-only dataset, these discontinuities are largely excluded already. Supplementary Fig. 2 presents overlapping areas and their boundaries over the land regions.

Following Taylor (2001), the remaining discontinuities along the borders of overlapping areas on land were quantified using a Normalised Root Mean Square Difference (NRMSD) based methodology across the climate variables, scenarios, and domain interfaces^79^. The NRMSD were grouped into four standardized categories: Excellent, Good, Moderate, and Poor. Based on these metrics, all variables except for precipitation fell within the excellent category (NRSMD < 0.5). Precipitation was categorized as Good, showing a NRMSD slightly above 0.5. The comprehensive discontinuity analysis, including its details and results by model and dataset, is presented in the supplementary material (Supplementary Tables 1, 2, and 3 and Figs. 3, 4, and 5).

**Fig. 2:**
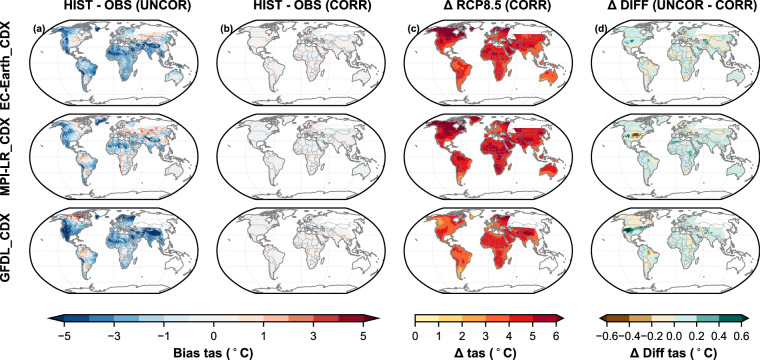
Mean surface air temperature (tas) biases over 1960-2019 against GSWP3-W5E5 v2.0 observational dataset and future changes between 2070-2099 and 1970-1999 under RCP8.5 scenario: column a) shows bias in raw CORDEX datasets, (column b) shows bias in GloBCORD-HD, (column c) shows change signal in GloBCORD-HD dataset, (column d) shows difference in change signal from raw CORDEX datasets and GloBCORD-HD. Each row corresponds to each of the downscaled CORDEX ESMs.

**Fig. 3:**
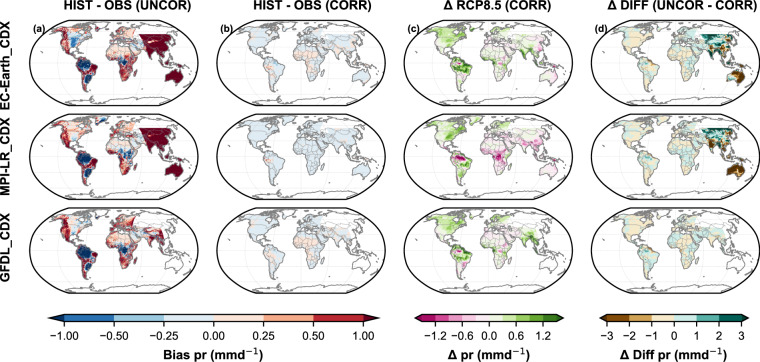
Same as Fig. 2, but for precipitation (pr).

**Fig. 4:**
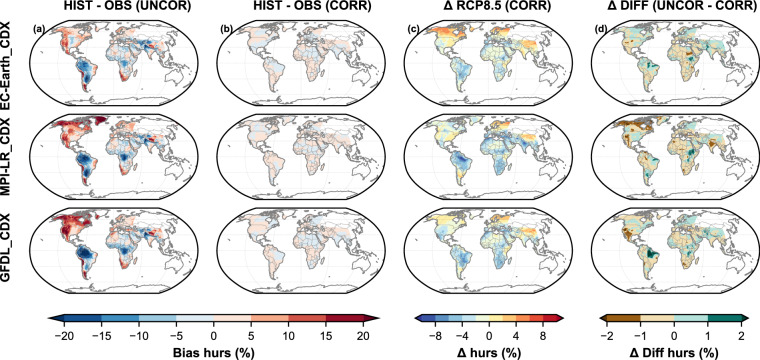
Same as Fig. 2, but for relative humidity (hurs).

**Fig. 5:**
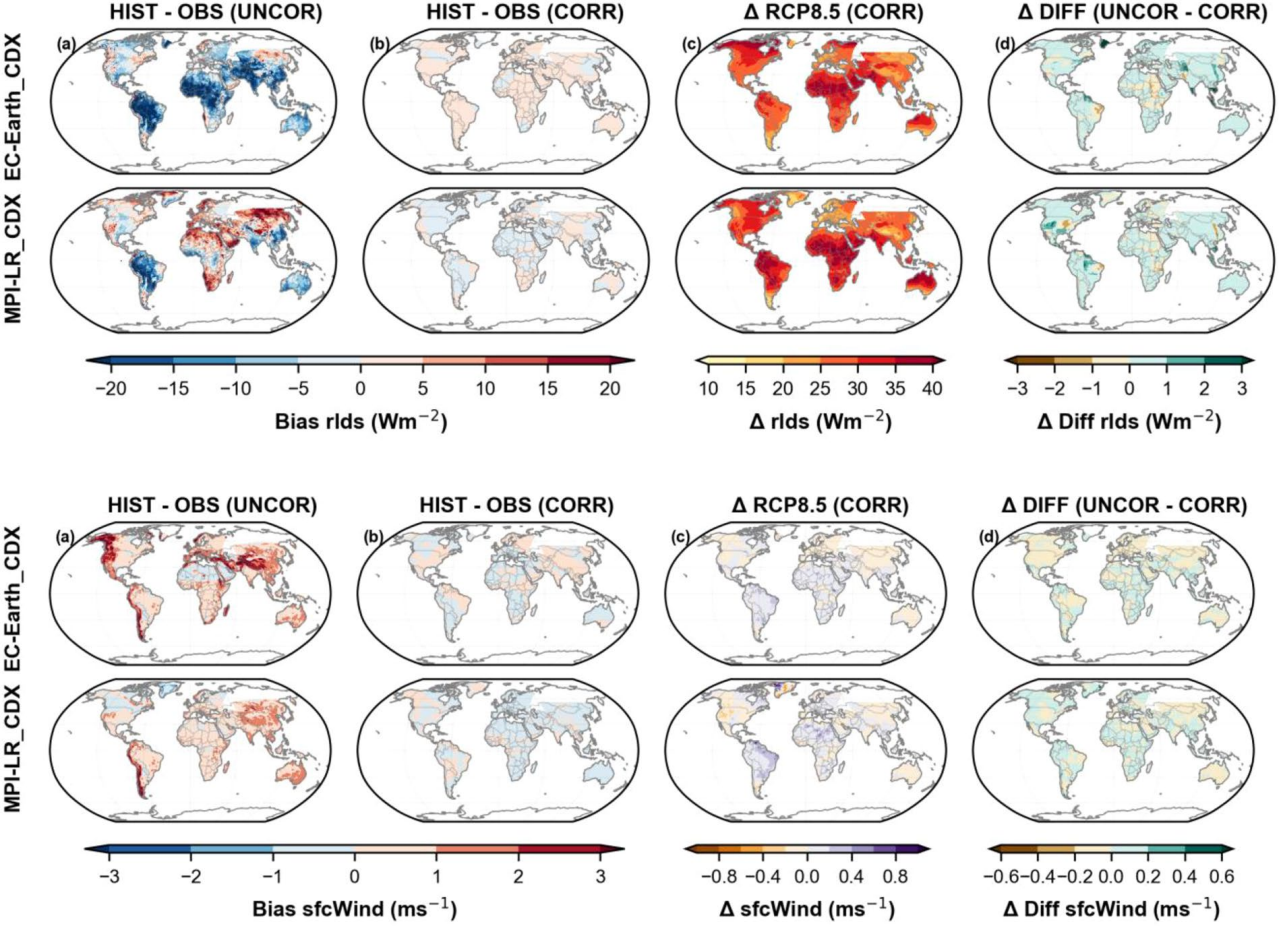
Same as Fig. 2, but for surface winds (sfcWind) and longwave radiation (rlds). Note: These variables are not available from CORDEX downscaled GFDL_CDX.

## Data Records

The GloBCORD-HD^40^ datasets are freely available on the Universität Hamburg’s Zentrum

 für Nachhaltiges Forschungsdatenmanagement using 10.25592/uhhfdm.18068. Alternatively, using 10.25592/uhhfdm.17395. This link represents all versions and will always resolve to the latest one. They include seven variables, i.e., tas, tasmax, tasmin, pr, hurs, rlds, and sfcWind for MPI-LR_CDX, EC-EARTH_CDX, whereas the first five variables are for GFDL_CDX. These datasets contain the historical period of 1950–2019 (70 years) for EC-EARTH_CDX and GFDL_CDX, and 1960–2019 (60 years) for the MPI-LR_CDX. The future period in the GloBCORD-HD^40^ datasets spans from 2020 to 2099 and contains climate projections from all three RCP scenarios, except RCP2.6 for the GFDL_CDX. It consists 71 datasets in all, i.e., 28 each for MPI-LR_CDX and EC-EARTH_CDX, and 15 for GFDL_CDX, with an average of ~4 GB per variable per scenario and a total of about ~275.5 GB for all.

All the bias-corrected datasets were stored in a self-describing netCDF format. The netCDF files are named as “VariableAdjust_GBC-50_ESM_scenario_RCM_GSWP3-W5E5-yy-yy_day_yyyy-yyyy.nc,” where “VariableAdjust” refers to individual variables, e.g., tasAdjust for tas, prAdjust for pr, and hursAdjust for hurs. The expression “GBC-50” refers to the Global Bias-Corrected CORDEX datasets at Half Degree (0.5°) resolution (GloBCORD-HD^40^), and “ESM” refers to the driving ESM used. The “Scenario” denotes the RCP2.6, RCP4.5, or RCP8.5 experiments. The “RCM” in the file names refers to the name of RCMs that downscaled the same ESM and were used to form the global dataset. The “GSWP3-W5E5-yy-yy” represents the observational dataset, the start and end years of the period used for bias correcting the CORDEX^12^ dataset, depending on their overlapping lengths. The expression “yyyy-yyyy” denotes the period of the data.

Since the historical simulations were extended using the RCP8.5 data for the period 2006–2019, their scenario name is replaced with the RCP8.5 following the guidelines of the Data Reference Syntax (DRS) for bias-adjusted CORDEX simulations format^80^. Hence, the historical and the RCP8.5 experiments cannot be differentiated based on the scenario name but based on the periods. The new corrected historical period, appended with RCP8.5 data, starts either from 1950 or 1960 and ends in 2019, whereas the future scenario of RCP8.5 starts from 2020 and ends in 2099. A technical summary of all the variables can be found in Table 2.

**Table 2:** Technical Summary for variables in the GloBCORD-HD dataset.

Parameter	EC-EARTH_CDX	MPI-LR_CDX	GFDL_CDX*
Variable name	tasAdjust, tasminAdjust, tasmaxAdjust, prAdjust, hursAdjust, rldsAdjust*, sfcWindAdjust*		
Standard Variable & Units	tas, tasmax, tasmin, pr, hurs, rlds, sfcWind K, K, K, kg m^−2^ s^−1^, %, Wm^−2^, ms^−1^		
Variable Description	Near-Surface Air Temperature, Minimum Near-Surface Air Temperature, Maximum Near-Surface Air Temperature, Precipitation, Near-Surface Relative Humidity, Surface Downwelling Longwave Radiation, Near-Surface Wind Speed		
ECS	~3.2 K	~3.6 K	~2.4 K
Historical Period	1950-2019	1960-2019	1950-2019
Future Period	2020-2099		
RCP Scenarios	RCP2.6, RCP4.5, RCP8.5		RCP4.5, RCP8.5
Spatial & Temporal Resolution	0.5° × 0.5° (~50 km) Daily		
Spatial Coverage/Grid Type	167°W to 180°E, 56°S to 76°N (Maximum) Regular latitude-longitude		
Number of Files	28		15
File Format	NetCDF		
Total Data Volume	~137.7 Gb	~130.6 Gb	~55.5 Gb
Correction Method	ISIMIP3BASD v2.5		
Reference Dataset	GSWP3-W5E5 v2.0 (1950-2019)		

Note:* rlds and sfcWind were not available for GFDL_CDX.

## Technical Validation

### Spatial validation

Since we used the GSWP3-W5E5 v2.0^45,46^ dataset for bias correction, despite its own biases, it is our “target” dataset against which we assess the performance of our bias-corrected GloBCORD-HD^40^ datasets. Each plot provides spatial, temporal, and statistical insights into the climate variables before and after correction, offering a comprehensive analysis of the GloBCORD-HD^40^ dataset’s accuracy and its applicability for climate impact assessments. The spatial representations show effective correction across different geographical regions and climate zones, validating the dataset’s robustness for global climate impact assessments. Historical biases were calculated as the difference between the simulated and the observed climates for the 1960–2019 period. The climate change signal was calculated for both bias-corrected and raw datasets as the difference between their means of 2070–2099 and 1970–1999 (30 years). The difference in the climate change signals from the raw and corrected datasets also suggests their agreement and effectiveness for onward use in impact assessment.

Figure 2 shows warm biases in the MPI-LR_CDX but cold biases in the EC-EARTH_CDX and GFDL_CDX, particularly over the West Asia domain. The models exhibited significant cold biases in temperature (tas) over various regions. Specifically, EC-EARTH_CDX and GFDL_CDX showed cold biases over Africa, West Asia, and the western part of North America. The MPI-LR_CDX model showed a slightly warmer bias over central to southern Africa and central Asia, but a slightly colder bias over northern Africa and the western part of North America. GFDL_CDX presented a larger cold bias over central North America and the West Asia domains, with the colour gradient indicating biases of up to 5 °C colder than observed data. The bias-corrected temperature maps showed marked reductions in these discrepancies. We noted that the temperature change from bias-corrected RCP8.5 is lower in GFDL_CDX than in the other two models. The climate signal difference map (Δ RCP8.5 Diff) shows the differences in the climate change signal between the raw dataset and the corrected dataset, showing stark agreement as their difference remains mostly around zero, except over the North America domain for MPI-LR_CDX and GFDL_CDX. Localized areas such as parts of Africa, South America, and North America showed slight changes in the signal, ranging within ±0.5°. This variation is consistent with Spuler *et al*. (2023), who found that even trends preservation corrections like ISIMIP3BASD v2.5^47^ can lead to some slight localised variations due to its use of empirical Cumulative Density Functions (CDFs) for trend mapping^29^. Dosio (2016) also found that bias correction altered the climate signal by up to ±0.7 °C across Europe^38^. Nonetheless, the bias correction demonstrated a notable effectiveness in reducing the systematic cold biases in EC-EARTH_CDX and GFDL_CDX simulations, and warm biases in MPI-LR_CDX, particularly in regions with complex terrain. The persistent cold bias exceeding 5 °C below zero at some locations over the West Asia domain in EC-EARTH_CDX and GFDL_CDX was simulated by RCA4 RCM. Such cold bias has successfully been minimized to within ±0.5 °C after correction. This improvement is particularly significant given the region’s complex topography and arid climate, which traditionally challenge climate model performance^4^. The effectiveness in correcting these bias patterns suggests that the ISIMIP3BASD^48^ has successfully managed diverse error structures across different climate regimes.

For precipitation as shown in Fig. 3, the raw historical simulations exhibited substantial wet and dry biases, particularly in tropical and high-latitude regions for all models. Notably, all three models show pronounced dry biases over southern South America and central Africa, and wet biases over western North America, southern Africa, Europe, and central South America. Stark wet bias (above 50 mmd^−1^) in MPI-LR_CDX and EC-EARTH_CDX over EAS and AUS domains downscaled by CCLM5-0-2 and CCLM4-8-17-CLM3-5, respectively, align with known challenges in simulating monsoon systems^3,4^. The overall precipitation biases were lower for the other domains. The bias correction effectively removes these discrepancies, bringing the precipitation estimates closer to observed values. The remaining residual biases appear minimal in most regions, suggesting that the correction approach successfully improves the models’ fidelity. Projected precipitation changes under the corrected RCP8.5 scenario indicate an overall increase in precipitation, particularly in the Amazonian belt and monsoon-dominated regions such as south and southeast Asia in all models. This aligns with expectations of an intensified hydrological cycle due to increased atmospheric moisture in a warming climate^81^. However, differences exist in the magnitude and spatial extent of these changes among models, which could be related to the model structure^82^. EC-EARTH_CDX and GFDL_CDX marked wetting of the Americas, west and southeast Asia, and western central Africa, whereas MPI-LR_CDX featured a moderate drying of Africa, the Amazon, Asia, and Australia. The differences in the RCP8.5 change signal between raw data and bias-corrected are particularly pronounced over East Asia and parts of the Austrasia, where the model simulations showed significant overestimations for precipitation for EC-EARTH_CDX and MPI-LR_CDX. Maraun (2016) noted that trend-preservation of a bias correction depends on the climate model simulating the right long-term climate signal. Drying signal after bias correction suggests inflated climate change signals in raw datasets^30^. This is consistent with the finding that bias correction can either increase or decrease the climate signal if there is too little or too much variability^82^. The correction of substantial biases in monsoon-dominated regions (>50 mmd^−1^) is a significant achievement. The huge overestimations, particularly observed from the CCLM4-8-17-CLM3-5 and CCLM5-0-2 RCMs over the Australasian and East Asian domains, were effectively reduced to within ±1 mmd^−1^ in most locations. However, residuals are noted in areas of extreme topographic variation and along coastlines, indicating limitations in the bias correction approach. Consistent with other studies, the bias correction slightly modified the climate change signal as seen in the EAS and AUS domains^82^. This can be attributed to the huge overestimations that impacted the raw simulations from these RCMs, thereby propagating into the difference. Such severe systematic errors, like in EAS and AUS, suggest that the underlying physical processes driving precipitation patterns were inadequately captured, either due to an error in production or during post-production processing. This casts doubt on the models’ projection of realistic future climate trajectories in these regions. This finding has important implications for users of our dataset, highlighting the need for cautious interpretation of projections in these regions, especially when conducting cross-domain analyses.

In Fig. 4, the humidity bias plots reveal significant overestimations (red) in North America, the south-western part of South Africa, and over the Himalayas, with pronounced underestimation (blue) in South America and parts of central Africa before correction, with biases ranging from −20% to +20%. The pattern is consistent across all three models (EC-EARTH_CDX, MPI-LR_CDX, and GFDL_CDX), though with varying intensity. After correction, these pronounced regional discrepancies largely disappear, demonstrating effective bias removal across all climate models. The corrected RCP8.5 projections show moderate humidity increases of 4–8% in the northern latitudes, eastern North America, and parts of Africa, with some tropical and mid-latitude regions experiencing decreases of similar magnitude. This largely agrees with findings from Fisher and Knutti (2013) about models projecting greater warming also showing a greater reduction in relative humidity^83^. The projected overestimation could be attributed to the cold biases projected in the models. The difference between the change signals is minor (±2%), indicating no alteration of the climate change signal, though some light regional variations exist.

Downward longwave radiation (rlds) in Fig. 5, exhibits substantial negative biases (blue, up to −20 Wm−²) in tropical regions before correction, particularly in South America, Africa, and parts of central to west Asia for the EC-EARTH_CDX model, while the MPI-LR_CDX model shows contrasting patterns with significant positive biases (10–20 Wm−²) over central Asia, Europe, and Africa. This aligns with the findings of Graeme *et al*. (2012), that climate models are biased up to about 10–20 Wm^−^^2^ ^84^. Post-correction maps display dramatically reduced biases globally, with most regions showing values remarkably close to observational data, suggesting the correction methodology effectively addresses model-specific systematic errors. The corrected RCP8.5 projections reveal strong and consistent increases in downward longwave radiation (10–40 Wm−²) across the globe, with particularly intense warming signals in the northern latitudes, central Africa, and parts of South America, a pattern consistent with enhanced greenhouse effect under climate change. The magnitude of these changes significantly exceeds the original bias in many regions, indicating a robust climate change signal. Differences between corrected and raw remain small (±3 Wm−²) but showed coherent spatial patterns, suggesting regional sensitivity to the bias correction methodology.

Surface wind (sfcWind) biases (also shown in Fig. 5) show a distinct pattern of overestimations (red, up to 3 ms−¹) along coastal regions before correction, especially western North America, western South America, and parts of West Asia, with underestimation (blue, down to −2 ms−¹) over the Sahara for EC-EARTH_CDX. The MPI-LR_CDX model showed a slightly lower overestimation across most domains except the western part of South America, which exhibited up to 2–3 ms−¹ overestimation. These patterns suggest systematic model difficulties in representing coastal wind dynamics and topographic effects. The corrected maps demonstrate successful bias reduction, with values approaching zero across most regions, though subtle residual patterns remain visible. Projected changes under RCP8.5 are modest compared to other climate variables, within ±0.8 ms−¹, with slight increases in some mid-latitude regions and decreases in parts of the tropics. The difference between correction methods is minimal (±0.6 ms−¹), indicating that for wind speeds, the choice of bias correction approach has limited influence on climate change projections, though the spatial coherence of these differences suggests systematic rather than random effects of bias correction.

## Temporal Validation

**Fig. 6:**
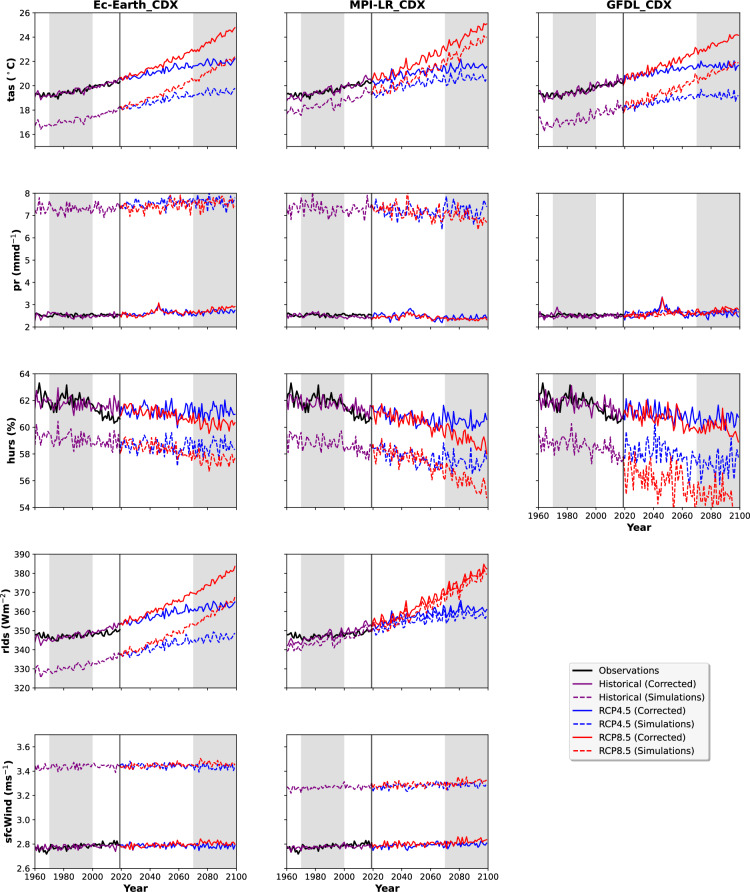
Time series of spatial means of tas, pr, hurs, rlds, and sfcWind considering AFR, SAM, WAS, EUR, and MNA Domains together for all three models. Each column represents each dataset from a particular downscaled ESM, whereas each row presents individual variables from a particular dataset. The black line divides the historical period 1960–2019 from the future period 2020–2099. Historical observations and model simulations are shown in black and magenta, whereas future projections for RCP4.5 and RCP8.5 are presented in blue and red, respectively. The shaded areas represent the last three decades of the 20th (1970–1999) and the 21st (2070–2099) centuries. Dashed lines present raw data, whereas solid lines present bias-corrected data.

Figure 6 reveals distinct mean spatial biases for each model and model-specific responses to bias correction across the historical period and future projections. For consistency, spatial means are calculated over five corrected CORDEX^12^ domains *(AFR, EUR, SAM, MNA, WAS)*, available in all datasets and variables for all historical, RCP8.5, and RCP4.5. For temperature, all three raw datasets exhibited consistent cold biases of about 2-3 °C, with EC-EARTH_CDX and GFDL_CDX featuring higher cold biases than MPI-LR_CDX. The Bias correction aligned the raw data well with the GSWP3-W5E5 v2.0 dataset, where mean biases were reduced to about <0.1 °C. The analysis focuses on the accuracy of historical simulations relative to observations and the implications of bias correction for future projections under the RCP8.5 and RCP4.5 scenarios. While raw data from all models’ project warming under both RCP scenarios, the bias correction adjusted the mean bias in EC-EARTH_CDX and GFDL_CDX projections by approximately +2 to +3 °C by 2099 under RCP8.5, compared to only +1 °C in MPI-LR_CDX. This difference suggests that the historical bias structures partially propagate into the future climate signals, with larger historical biases resulting in more substantial adjustments to projected changes.The RCP8.5 scenarios projected a higher warming compared to RCP4.5 in all models. By the end of the century, warming is higher in MPI-LR_CDX  compared to the other two models, confirming the models’ high ECS.

The precipitation response to bias correction reveals critical challenges in climate modeling. The substantial wet bias in EC-EARTH_CDX and MPI-LR_CDX (approximately double the observed baseline) primarily originates from CCLM4-8-17-CLM3-5 and CCLM5-0-2 RCM simulations in the AUS and EAS domains, respectively. The exclusion of problematic domains in the GFDL_CDX results in markedly better performance, suggesting that domain-specific validation is essential before incorporating them into the global grid. Besides a minute increase, a notable feature is the minimal divergence between RCP scenarios for precipitation across all models, contrasting sharply with temperature responses.

**Fig. 7:**
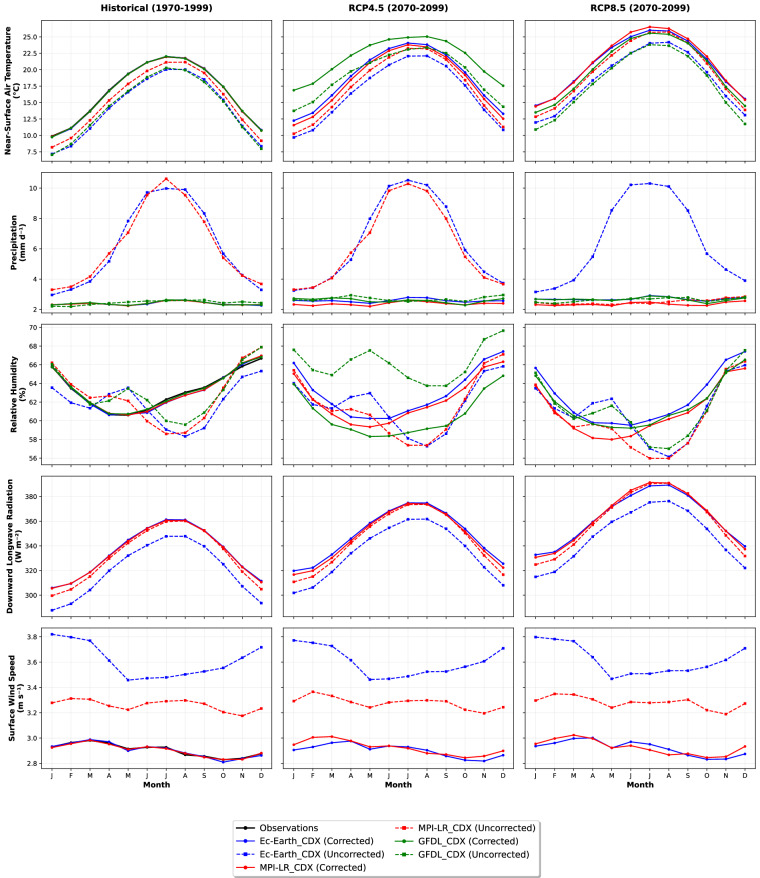
Monthly climatology of tas, pr, hurs, rlds, and sfcWind considering the mean of AFR, SAM, WAS, EUR, and MNA domains for the historical period (1970–1999), RCP4.5 (2070–2099), and RCP8.5 (2070–2099).

Relative humidity shows an underestimation of 2–3% for all datasets. It features a decreasing trend within historical and future periods, where such a decrease is higher in RCP8.5 than in RCP4.5 for the end-of-century climate, consistent with thermodynamic expectations in a warming atmosphere. Post-correction, there is a strong historical alignment with the observations for relative humidity. Surface wind speed simulations overestimated observations by approximately 0.5–0.7 m/s. The bias correction effectively aligns the projections with the observed values, but no discernible long-term trends are evident across all RCP scenarios, just like in precipitation. In contrast, downward longwave radiation is underestimated by 10–20 Wm^-^² in the raw simulations, reflecting the presence of the cold temperature bias in the models. The bias correction successfully aligned the historical radiation values with the observations. The future projections showed scenario-dependent increases, with RCP8.5 yielding a 30–40 Wm^-^² rise by 2099 and with a moderate increase for RCP4.5. This trend aligns with expectations under enhanced greenhouse forcing. The monthly climatologies for tas, pr, hurs, rlds, and sfcWind are shown in Fig. 7 for historical (1970–1999), RCP4.5, and RCP8.5 for 2070–2099.

### Validation insights

Table 3 shows a statistical evaluation of the divergence between pre-2006 and post-2006 data to determine whether appending RCP8.5 data (2006–2019) to extend the historical period degraded the model-observation agreement. We used mean bias, Mean Absolute Error (MAE), and correlation (Corr) measures for both the 1960–2005 and 2006–2019 periods against the observations from 1960–2019. All metrics remained stable between pre- and post-2006, which supports the validity of extending the historical period. This indicates that the use of the RCP8.5 data did not introduce significant additional errors, at least for these variables and models assessed.

**Table 3:** Evaluation of pre-2006 and post-2006 bias correction periods to quantify the impact of the appended RCP8.5 data as part of the historical scenario.

Model (Variable)	Bias		MAE		Corr	
	Pre-2006	Post-2006	Pre-2006	Post-2006	Pre-2006	Post-2006
EC-EARTH_CDX (tas)	−0.012	0.109	0.190	0.155	0.776	0.446
MPI-LR_CDX (tas)	−0.045	0.138	0.197	0.240	0.746	0.576
GFDL_CDX(tas)	−0.050	0.115	0.218	0.197	0.747	0.477
EC-EARTH_CDX (pr)	−0.029	−0.003	0.090	0.079	0.774	0.774
MPI-LR_CDX (pr)	−0.004	−0.006	0.094	0.111	0.085	0.190
GFDL_CDX(pr)	−0.023	0.007	0.097	0.069	0.110	−0.030
EC-EARTH_CDX hurs	−0.209	0.183	0.515	0.853	0.919	0.472
MPI-LR_CDX hurs	−0.179	0.614	0.422	0.627	0.496	0.342
GFDL_CDX(hurs)	−0.157	0.588	0.514	0.682	0.172	0.406
EC-EARTH_CDX (rlds)	−0.375	2.653	1.330	2.653	0.451	0.453
MPI-LR_CDX (rlds)	−0.883	2.804	1.726	2.828	0.469	0.372
EC-EARTH_CDX (sfcWind)	0.002	−0.017	0.020	0.023	0.547	−0.100
MPI-LR_CDX (sfcWind)	0.002	−0.017	0.020	0.023	0.547	−0.100

**Fig. 8:**
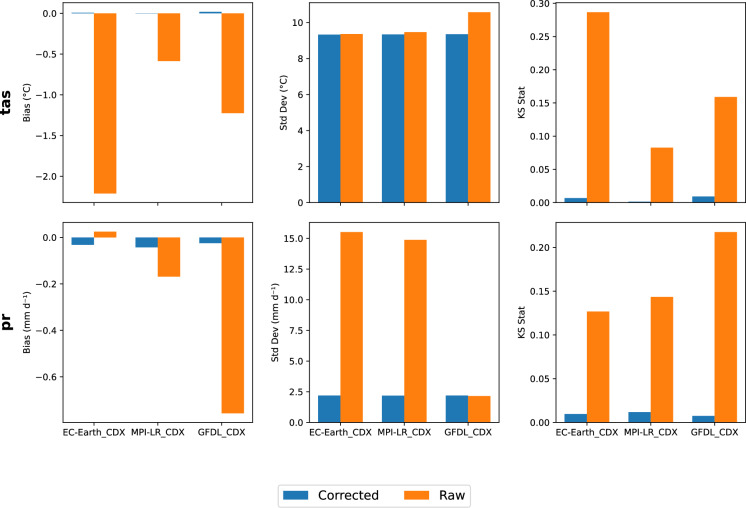
Comparison of statistical measures for the corrected and raw historical (1960–2019) tas and pr for the three ESMs of EC-EARTH_CDX, MPI-LR_CDX, and GFDL_CDX, considering the average of five domains (AFR, SAM, WAS, EUR, and MNA). Statistical measures include: (left column) mean bias, (middle column), standard deviation, and (right column), Kolmogorov-Smirnov statistics (KS Stat).

In Fig. 8, the statistical measures for the corrected and raw historical (1960–2019) tas and pr for the three ESMs of EC-EARTH_CDX, MPI-LR_CDX, and GFDL_CDX are compared, considering the average of five domains (AFR, SAM, WAS, EUR, and MNA). Statistical measures include: (left column) mean bias, (middle column) standard deviation, and (right column) Kolmogorov-Smirnov statistics (KS Stat)^85^. The statistical metrics provide quantitative evidence to complement our spatial and temporal analyses. The differential response across variables and metrics offers important insights into the nature of model biases and correction efficacy. The statistical metrics are calculated using spatial samples from climatological mean fields.

The bias correction significantly improved the raw datasets, as shown for both precipitation (pr) and temperature (tas). These evaluations allow us to assess not only the correction of mean state biases but also improvements in variability and extreme events, providing a more comprehensive validation of the correction methodology. These improvements have important implications for the reliability of climate projections and their application in impact studies. For precipitation, correlations are improved significantly by approximately 80% (from 15 to around 2.5 mm/day) for EC-EARTH_CDX and MPI-LR_CDX, representing a substantial improvement in overall accuracy. Similar improvements of approximately 30–50% were observed over the temperate zones in Spuler *et al*. (2023), reinforcing the robustness of these statistical corrections across diverse climates. A similar improvement is observed in the Kolmogorov-Smirnov statistic (KS stat)^85^, which decreases from between 0.12–0.21 to nearly 0.01. This pronounced improvement in distributional agreement is crucial for applications focused on hydrological extremes, as it indicates that the corrected data better captures not only mean conditions but also the frequency and intensity of precipitation events across the probability distribution^86^. For temperature, the substantial KS stat improvements (particularly in EC-EARTH_CDX, from 0.29 to 0.01) indicate that the correction method transformed a poor distributional match into an almost excellent one. This has important implications for impact studies focused on temperature extremes, as corrected data more accurately represent the frequency and intensity of heat waves and cold spells that often drive climate impacts.

The analysis highlights several broader implications. First, the effect of bias correction is variable-dependent, with more pronounced improvements for precipitation error metrics, suggesting inherent differences in how climate models simulate these variables. Second, model-specific responses to bias correction vary, with EC-EARTH_CDX showing the most significant improvement in both bias and distributional accuracy. Despite these variations, post-correction performance across all models converges, indicating that the bias correction promotes greater consistency in the multi-domain dataset. The results underscore the importance of evaluating both raw simulations and corrected model outputs when selecting models for specific applications. Overall, the bias correction improved the alignment between model outputs and observations for temperature, relative humidity, radiation, and wind speed (see supplementary Figs. 6–8), which exhibited substantial biases in raw datasets. For precipitation corrections, the biases were hugely dependent on the EAS and AUS datasets, which had significant biases in the raw simulations.

GloBCORD-HD^40^ offers several key advantages over existing bias-corrected CMIP5/6^18,87^ products, such as the ISIMIP3b^49^ and NEX-GDDP (https://registry.opendata.aws/nex-gddp-cmip6, last access: 12 February 2025), which have been statistically downscaled from the coarser GCMs. In contrast, GloBCORD-HD^40^ leverages outputs of the dynamically downscaled RCM that explicitly resolve regional physical processes, topographic effects, and land-surface interactions, providing a superior representation of local climate drivers, extreme events, and sub-daily variability, unlike GCMs. Secondly, GloBCORD-HD^40^ does not aim to provide higher-resolution datasets but to ensure the robust use of existing CORDEX^12^ dynamically downscaled datasets, particularly for impact studies. Hence, GloBCORD-HD^40^ provides, for the first time, a dynamically downscaled quasi-global half-degree dataset, which is consistently bias-adjusted using a sophisticated bias adjustment technique against a single observational dataset widely adopted by the impact assessment community. Therefore, GlobCORD-HD^40^ complements rather than competes with existing datasets of the same resolution.

Despite these qualities, GloBCORD-HD^40^ features several limitations. First, the quality of the bias correction fundamentally depends on the accuracy of the GSWP3-W5E5 v2.0^45,46^ observational dataset. Although it has undergone bias adjustments, it may still contain systematic errors that could have propagated to GloBCORD-HD^40^. Furthermore, the use of RCP8.5 as “pseudo-historical” only introduced a negligible impact for all variables, except for a minute difference for the rlds variable (Table 3). Owing to partial coverage of CORDEX^12^ experiments and their strict selection criteria, GloBCORD-HD^40^ provides only quasi-global coverage, unlike the CMIP5/6^18,87^ corrected products, which offer global coverage, including oceans. The CORDEX^12^ regional domains overlap over a few regions, as shown in Fig. 1 and Supplementary Fig. 1. Here, we encourage data users to exercise caution when using GloBCORD-HD^40^ from overlapping regions, as these represent the ensemble mean of the available datasets from more than one regional domain. Merging regional domains into a quasi-global dataset may also introduce discontinuities along the individual domain boundaries. Such discontinuities are minimal for the historical period, as we merge the individual regional domains only after bias-correcting them to a single continuous observational dataset. Discontinuities in future scenarios may arise from different change signals simulated by individual RCMs. These have also been smoothed out due to merging the overlapping areas using ensemble means. As shown in the Supplementary Fig. 1, a large part of these discontinuous boundaries lies over the oceanic region (e.g., the eastern and western boundaries of CAM, NAM, SAM, AFR, EUR, EAS, and AUS domains, and southern WAS and MNA, and southern and northern boundaries of EAS), which is already excluded from the GloBCORD-HD^40^ since it is a land-only dataset. Supplementary Fig. 2 shows the remaining overlapping areas and their boundaries over land regions. Our discontinuity analysis of the boundary areas showed seamless transition along the domain boundaries (Supplementary Tables 1, 2, and 3 and Figs. 3, 4, and 5). Nevertheless, we advise cautious use of data along domain boundaries.

Despite identified limitations, GloBCORD-HD^40^ offers the climate impacts community a valuable resource that bridges the gap between raw climate model outputs and the needs of regional-scale data by impact modelers and decision-makers. The combination of three RCP scenarios (2.6, 4.5, 8.5) from three ESMs spanning different ECS creates a comprehensive uncertainty matrix that addresses both forcing pathway uncertainty and model physics uncertainty. This approach ensures that GloBCORD-HD^40^ captures the wide breadth of possible future climate trajectories, from conservative warming projections (low ECS, RCP2.6) to more extreme scenarios (high ECS, RCP8.5), thereby providing impact modelers with a scientifically robust foundation for climate risk assessment across various sectors. By providing transparent documentation of both correction methodologies and validation results, we enable users to make informed judgments about this dataset for their specific research or planning context. The datasets represent a significant advancement in providing quasi-global bias-corrected dynamically downscaled CORDEX^12^ datasets at native resolution. Our comprehensive validation across spatial, temporal, and statistical dimensions demonstrates substantial improvements in model accuracy while preserving essential climate change signals. The corrected temperature data provides more accurate inputs for heat stress analyses and energy demand projections, while improved precipitation statistics strengthen flood risk assessments and water resource planning. These improvements directly translate to enhanced reliability for climate impact assessments. Furthermore, the multi-model, multi-scenario approach of GloBCORD-HD^40^ also allows users to better characterize projection uncertainties, a critical aspect for robust adaptation planning.

## Supplementary information

Supplementary Information

## Data Availability

The dataset is available at the Universität Hamburg’s Zentrum für Nachhaltiges Forschungsdatenmanagement using 10.25592/uhhfdm.18068. Alternatively, using 10.25592/uhhfdm.17395. This link represents all versions and will always resolve to the latest one.

## **Code availability**

The regridding of climate simulations to the reference dataset was performed using Climate Data Operator (CDO, version 2.4.1), which can be obtained from the Max Planck Institute of Meteorology at https://code.mpimet.mpg.de/projects/cdo. The study uses the Python implementation of the ISIMIP3BASD from the “ibicus” package, which can be downloaded at https://github.com/ecmwf-projects/ibicus. All sources of the employed datasets are open access and free of charge. However, registration may be required to acquire CORDEX experiments through ESGF websites. Figures are plotted in Python using the Matplotlib library. Bias-correction and post-processing scripts are available and can be accessed at 10.25592/uhhfdm.17395.
