# Supplementary material for: Global Bias-Corrected CORDEX Datasets at Half Degree Resolution
Source: Sci Data. 2025 Nov 12;12:1781. doi: 10.1038/s41597-025-06200-4 (PMC12612069; doi:10.1038/s41597-025-06200-4)
Supplement: Supplementary file 1 — Supplementary Information [file 41597_2025_6200_MOESM1_ESM.pdf]

Boundary Discontinuities in GloBCORD-HD

Following Taylor (2001), a Normalised Root Mean Square Difference (NRMSD) based methodology was used to quantify and evaluate discontinuities across multiple climate variables, scenarios, and domain interfaces[1]. This approach objectively quantifies boundary discontinuities, highlighting the consistency of the merger across the multiple CORDEX RCMs.

Boundary Detection and Construction

For each domain, we first calculated the RMSD between each boundary grid cell and its neighboring grid cell from the overlapping domains for all overlapping boundaries (eqn. 1). Then, the grid cell-based RMSD was averaged over each domain. The domain-averaged RMSD was then normalized by the averaged Standard Deviations (eqn. 2) of both overlapping domains, i.e., NRMSD (eqn. 3). Standard deviation for each domain was calculated from the pooled data from the boundary grid cells that participated in RMSD calculation.

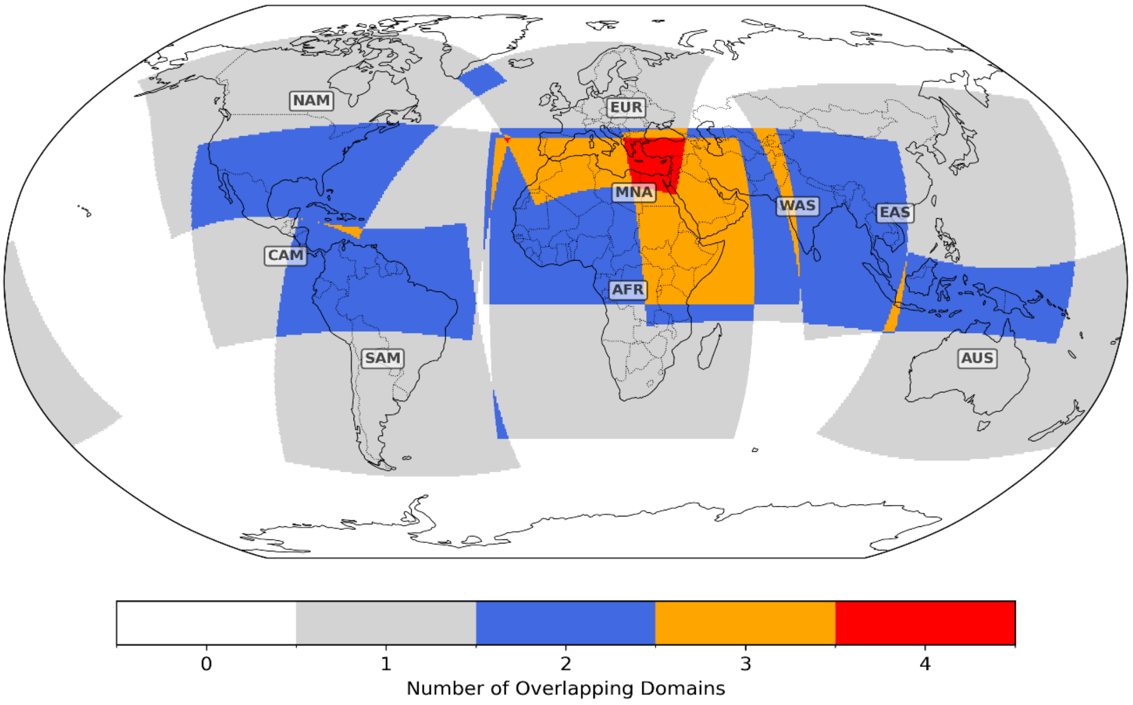

Figure 1. A global map showing regional climate model domain overlaps by the number of domains, including over oceans. Colours indicate the number of overlapping domains.

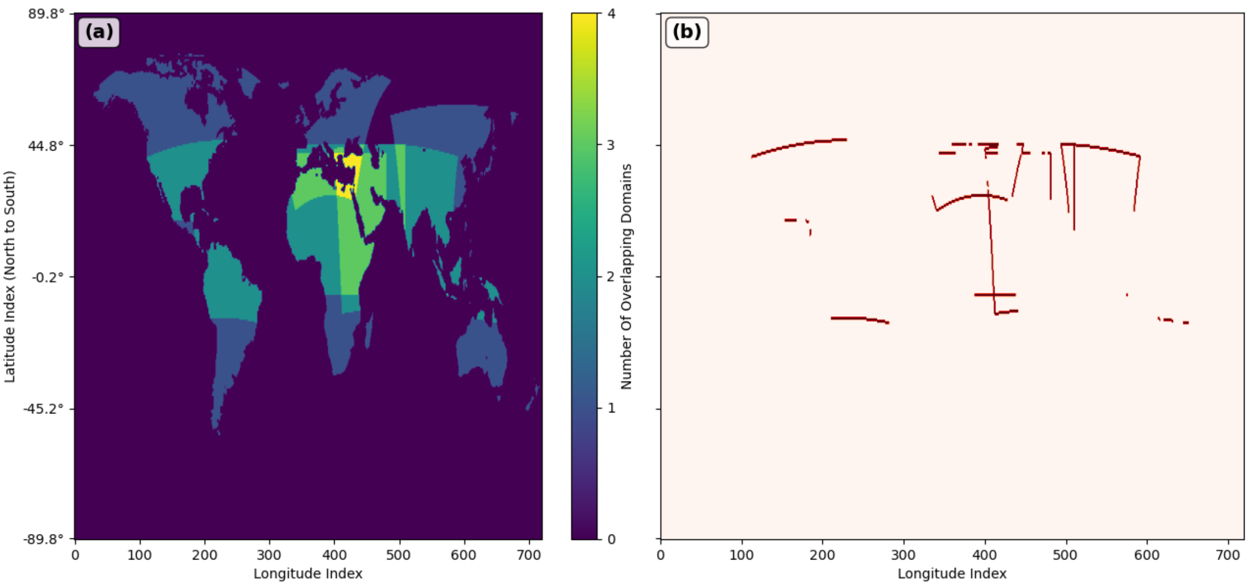

Figure 2. a). Same as Figure 1, but showing overlapping domains only over land. b). A map showing the boundary grid cell pairs with possible discontinuities in GloBCORD-HD.

$$RMSD = \sqrt{\frac{1}{T}\sum_{t=1}^{T}\left[X_{d_1}(t) - X_{d_2}(t)\right]^2} \quad \text{..............} \quad \textit{eqn.1}$$

$$Sd_n = \sqrt{\frac{1}{N-1}\sum_{i=1}^{N}\left(X_{d_n}(t) - \bar{X}_{d_n}\right)^2} \quad \text{..............} \quad \text{eqn.2}$$

$$\mathrm{NRMSD} = \frac{RMSD}{\sqrt{\left(\frac{\mathrm{Sd_1}^2 + \mathrm{Sd_2}^2}{2}\right)}} \quad \text{..............} \quad \text{eqn.3}$$

Where:

- T = Total *number of time steps*
- t = Time *index*
- $X_{d_n}(t)$ = Spatial mean (averaged across all boundary cells on domain n side) at each time step t
- Sd_n = Standard deviation of Data points
- $\bar{X}$ = Mean of data
- N = Total number of boundary segments/grid pairs
- n = Domain side (1 or 2)

We categorized the NRMSD into four categories of excellent, good, moderate, and poor based on the following criteria:

- NRMSD < 0.5 (Excellent): discontinuity is less than half the natural climate variability
- NRMSD 0.5-1.0 (Good): discontinuity comparable to natural variability
- NRMSD 1.0-2.0 (Moderate): discontinuity slightly exceeds natural variability
- NRMSD > 2.0 (Poor): discontinuity significantly exceeds natural variability

Results

The domain boundary discontinuities across all variables and RCP scenarios indicate that, GloBCORD-HD^2 exhibits seamless multi-domain integration. Except for precipitation, all other variables demonstrated an excellent boundary consistency (NRSMD < 0.5). Precipitation showed a good boundary consistency with NRSMD falling slightly above 0.5. The results show the suitability for regional/global climate analysis without significant impacts from discontinuities at domain interfaces.

Table 1. Table showing Discontinuity Statistic for EC-EARTH_CDX

Variable	Scenario	Mean RMSD	Std RMSD	Mean NRMSD	Std NRMSD	Quality	Grid cell Count
hurs	hist	3.901	2.157	0.303	0.193	EXCELLENT	854
hurs	rcp26	3.878	2.133	0.300	0.189	EXCELLENT	854
hurs	rcp45	3.886	2.131	0.297	0.184	EXCELLENT	854
hurs	rcp85	3.894	2.158	0.292	0.178	EXCELLENT	854
pr	hist	0.000	0.000	0.571	0.192	GOOD	1031
pr	rcp26	0.000	0.000	0.550	0.196	GOOD	854
pr	rcp45	0.000	0.000	0.597	0.223	GOOD	1031
pr	rcp85	0.000	0.000	0.594	0.224	GOOD	1031
tas	hist	1.410	1.121	0.261	0.426	EXCELLENT	1031
tas	rcp26	1.205	1.350	0.277	0.499	EXCELLENT	854
tas	rcp45	1.405	1.701	0.260	0.433	EXCELLENT	1031
tas	rcp85	1.408	1.693	0.237	0.362	EXCELLENT	1031
tasmax	hist	1.505	1.660	0.247	0.353	EXCELLENT	1031
tasmax	rcp26	1.292	1.337	0.247	0.385	EXCELLENT	854
tasmax	rcp45	1.509	1.649	0.240	0.325	EXCELLENT	1031
tasmax	rcp85	1.514	1.635	0.225	0.285	EXCELLENT	1031
tasmin	hist	1.581	1.767	0.264	0.329	EXCELLENT	1031
tasmin	rcp26	1.376	1.393	0.274	0.364	EXCELLENT	854
tasmin	rcp45	1.566	1.755	0.266	0.340	EXCELLENT	1031
tasmin	rcp85	1.570	1.754	0.252	0.316	EXCELLENT	1031
rlds	hist	7.976	7.621	0.213	0.221	EXCELLENT	1031
rlds	rcp26	8.236	8.007	0.223	0.234	EXCELLENT	947

rlds	rcp45	8.341	7.926	0.219	0.228	EXCELLENT	1031
rlds	rcp85	8.070	8.030	0.206	0.227	EXCELLENT	1031
sfcWind	hist	0.454	0.362	0.494	0.350	EXCELLENT	1031
sfcWind	rcp26	0.393	0.327	0.439	0.299	EXCELLENT	854
sfcWind	rcp45	0.454	0.361	0.493	0.351	EXCELLENT	1031
sfcWind	rcp85	0.454	0.360	0.494	0.352	EXCELLENT	1031

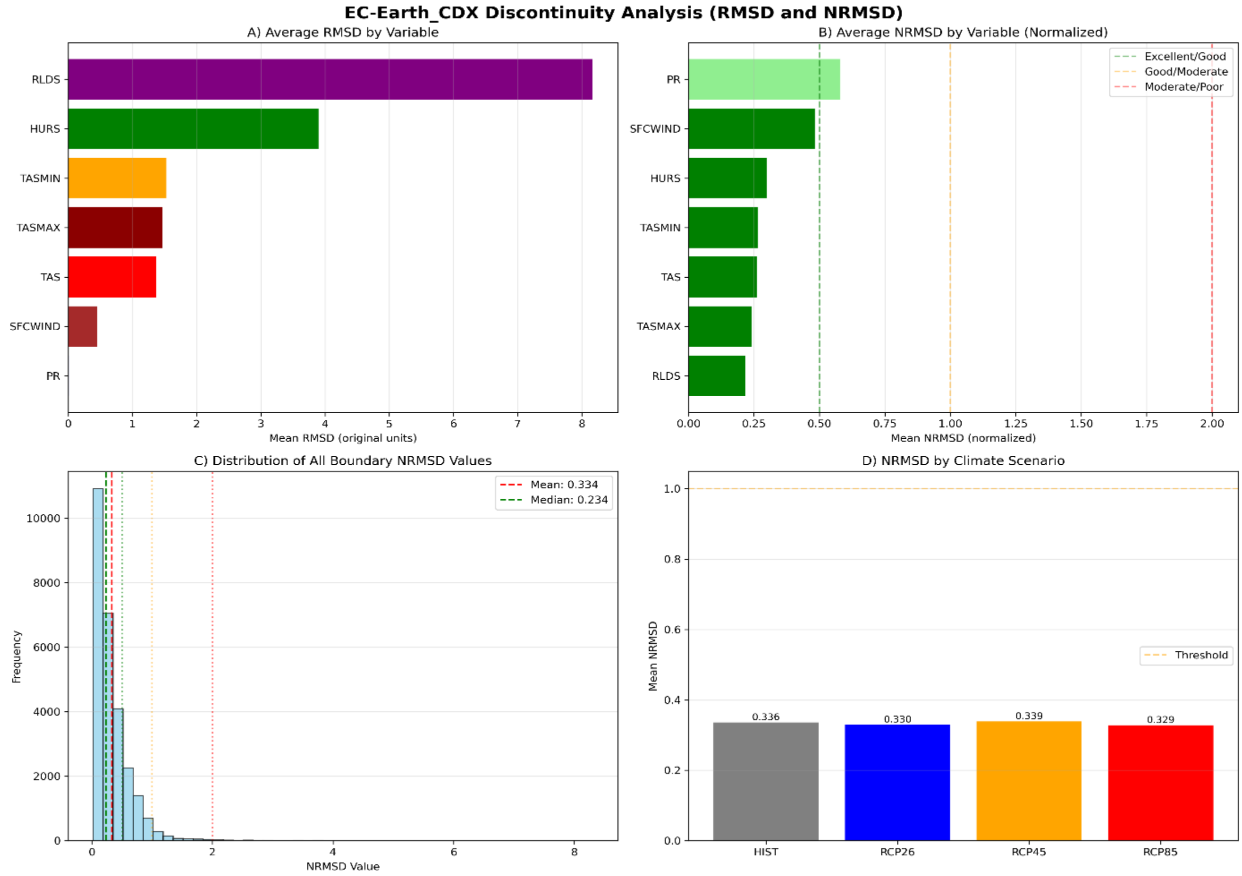

Figure 3. Showing the Discontinuity analyses for EC-Earth_CDX

Table 2. Table showing Discontinuity Statistics for MPI-LR_CDX

Variable	Scenario	Mean RMSD	Std RMSD	Mean NRMSD	Std NRMSD	Quality	Grid cell Count
hurs	hist	4.001	2.100	0.301	0.181	EXCELLENT	854
hurs	rcp26	3.947	2.277	0.297	0.209	EXCELLENT	724
hurs	rcp45	3.998	2.201	0.298	0.198	EXCELLENT	854
hurs	rcp85	4.020	2.235	0.295	0.197	EXCELLENT	854
pr	hist	0.000	0.000	0.558	0.178	GOOD	1031
pr	rcp26	0.000	0.000	0.589	0.166	GOOD	724
pr	rcp45	0.000	0.000	0.598	0.223	GOOD	1031
pr	rcp85	0.000	0.000	0.583	0.191	GOOD	1031

tas	hist	1.401	1.705	0.254	0.416	EXCELLENT	1031
tas	rcp26	1.207	1.424	0.292	0.495	EXCELLENT	724
tas	rcp45	1.434	1.729	0.254	0.393	EXCELLENT	1031
tas	rcp85	1.404	1.697	0.223	0.342	EXCELLENT	1031
tasmax	hist	1.555	1.640	0.249	0.334	EXCELLENT	1031
tasmax	rcp26	1.356	1.379	0.271	0.367	EXCELLENT	724
tasmax	rcp45	1.550	1.619	0.241	0.312	EXCELLENT	1031
tasmax	rcp85	1.555	1.608	0.223	0.272	EXCELLENT	1031
tasmin	hist	1.570	1.754	0.258	0.326	EXCELLENT	1031
tasmin	rcp26	1.346	1.470	0.284	0.384	EXCELLENT	724
tasmin	rcp45	1.557	1.754	0.261	0.370	EXCELLENT	1031
tasmin	rcp85	1.567	1.777	0.265	0.497	EXCELLENT	1031
rlds	hist	7.685	7.368	0.201	0.213	EXCELLENT	1031
rlds	rcp26	7.874	8.258	0.216	0.240	EXCELLENT	817
rlds	rcp45	7.711	7.598	0.199	0.220	EXCELLENT	1031
rlds	rcp85	7.728	7.759	0.190	0.218	EXCELLENT	1031
sfcWind	hist	0.480	0.365	0.504	0.348	EXCELLENT	1031
sfcWind	rcp26	0.393	0.294	0.450	0.300	EXCELLENT	724
sfcWind	rcp45	0.480	0.364	0.502	0.348	EXCELLENT	1031
sfcWind	rcp85	0.480	0.363	0.502	0.345	EXCELLENT	1031

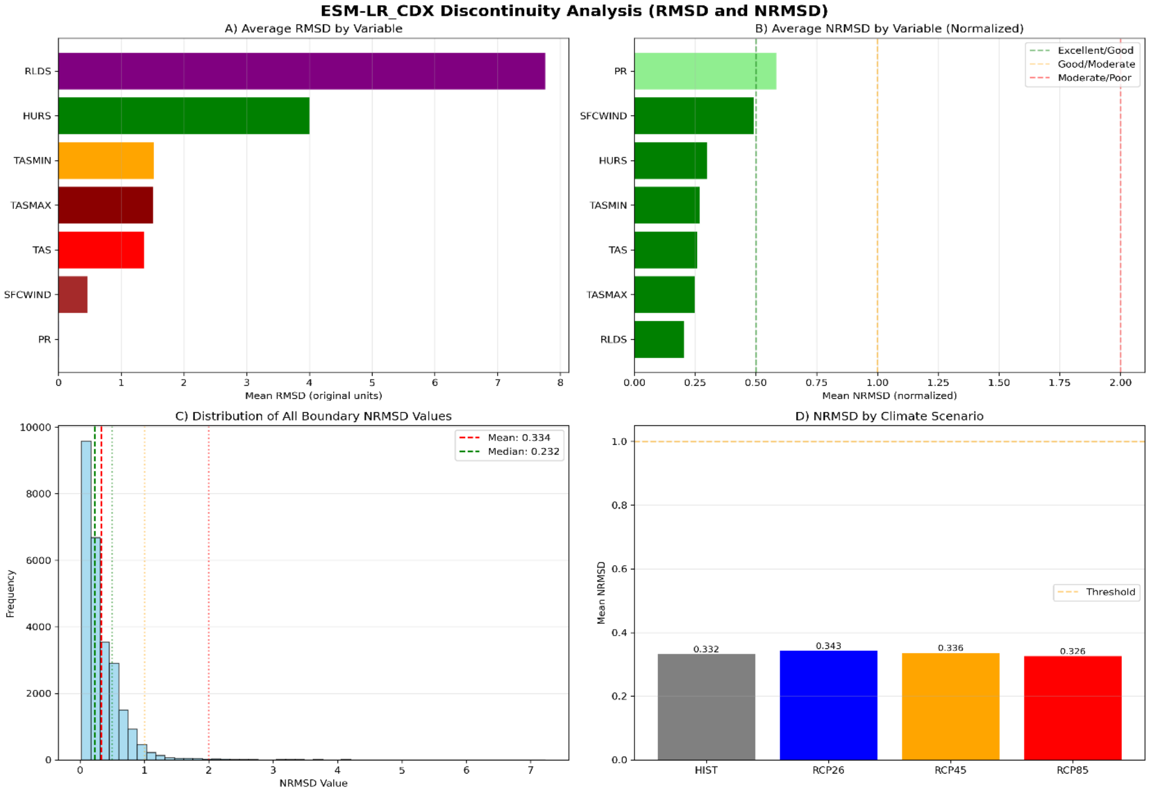

Figure 4. Same as Figure 3, but for ESM-LR_CDX

Table 3. Table showing Discontinuity Statistics for GFDL_CDX

Variable	Scenario	Mean RMSD	Std RMSD	Mean NRMSD	Std NRMSD	Quality	Grid cell Count
hurs	hist	3.964	2.200	0.309	0.198	EXCELLENT	854
hurs	rcp45	3.720	2.133	0.280	0.179	EXCELLENT	699
hurs	rcp85	3.952	2.205	0.293	0.178	EXCELLENT	854
pr	hist	0.000	0.000	0.668	0.216	GOOD	854
pr	rcp45	0.000	0.000	0.576	0.120	GOOD	699
pr	rcp85	0.000	0.000	0.644	0.205	GOOD	854
tas	hist	1.181	1.361	0.261	0.454	EXCELLENT	854
tas	rcp45	1.113	1.318	0.241	0.358	EXCELLENT	699
tas	rcp85	1.177	1.346	0.233	0.378	EXCELLENT	854
tasmax	hist	1.287	1.347	0.243	0.370	EXCELLENT	854
tasmax	rcp45	1.179	1.298	0.211	0.226	EXCELLENT	699
tasmax	rcp85	1.283	1.314	0.215	0.284	EXCELLENT	854
tasmin	hist	1.361	1.406	0.265	0.349	EXCELLENT	854
tasmin	rcp45	1.262	1.364	0.251	0.291	EXCELLENT	699
tasmin	rcp85	1.348	1.405	0.249	0.326	EXCELLENT	854

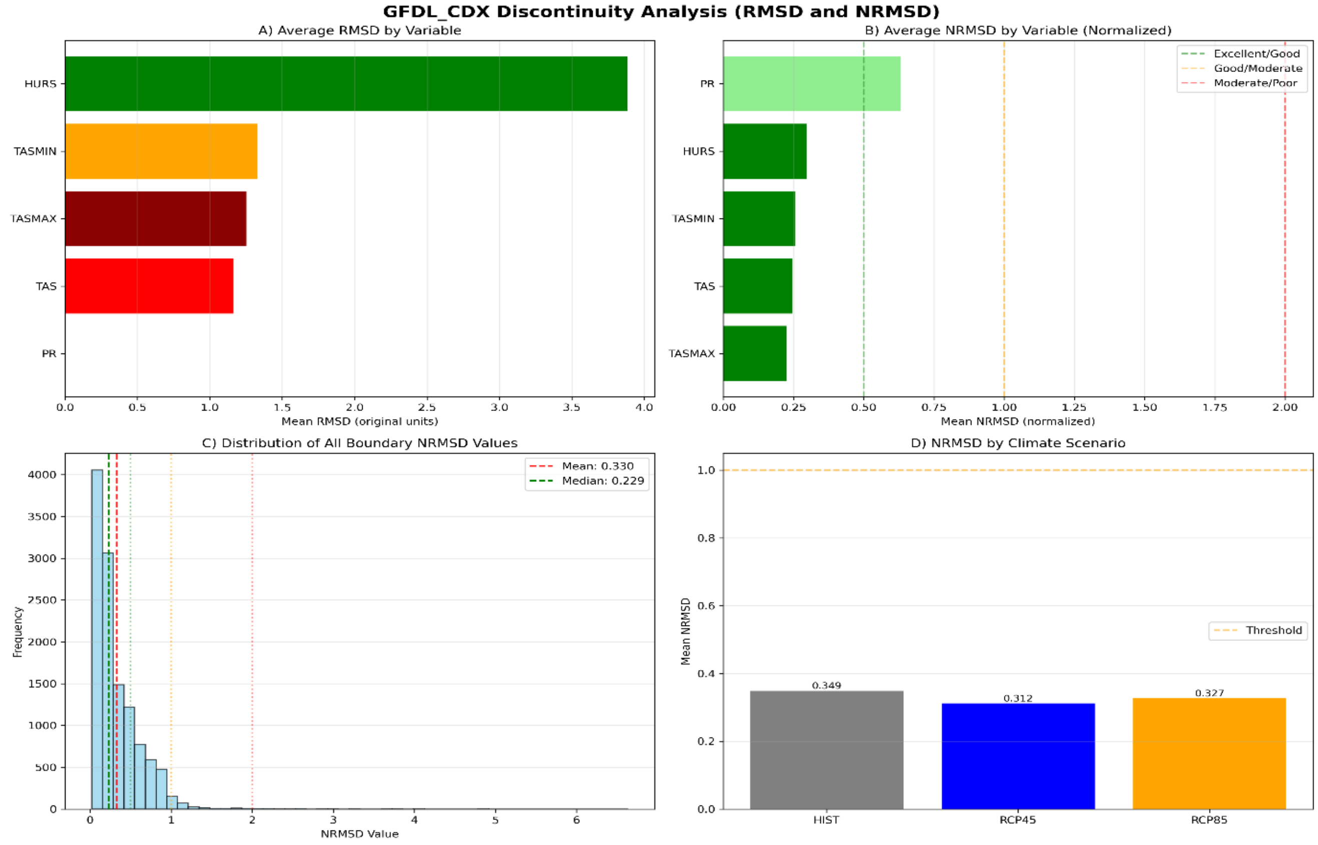

Figure 5. Same as Figure 3, but for GFDL_CDX

statistical measures

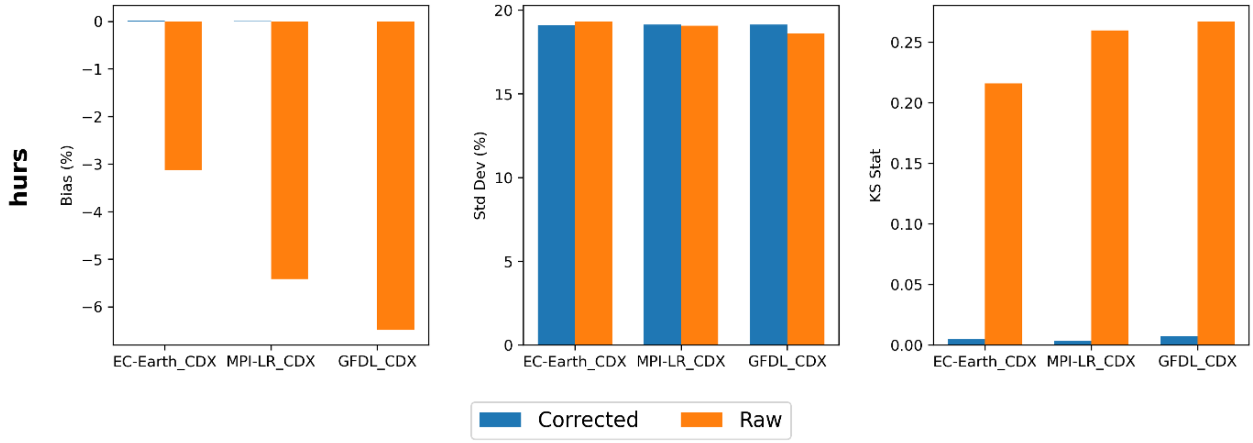

Figure 6. Comparison of statistical measures for the corrected and raw historical (1960-2019) hurs for the three CORDEX ESMs of EC-EARTH, MPI-ESM-LR, and GFDL-ESM2M, considering the average of five domains (AFR, SAM, WAS, EUR, and MNA).

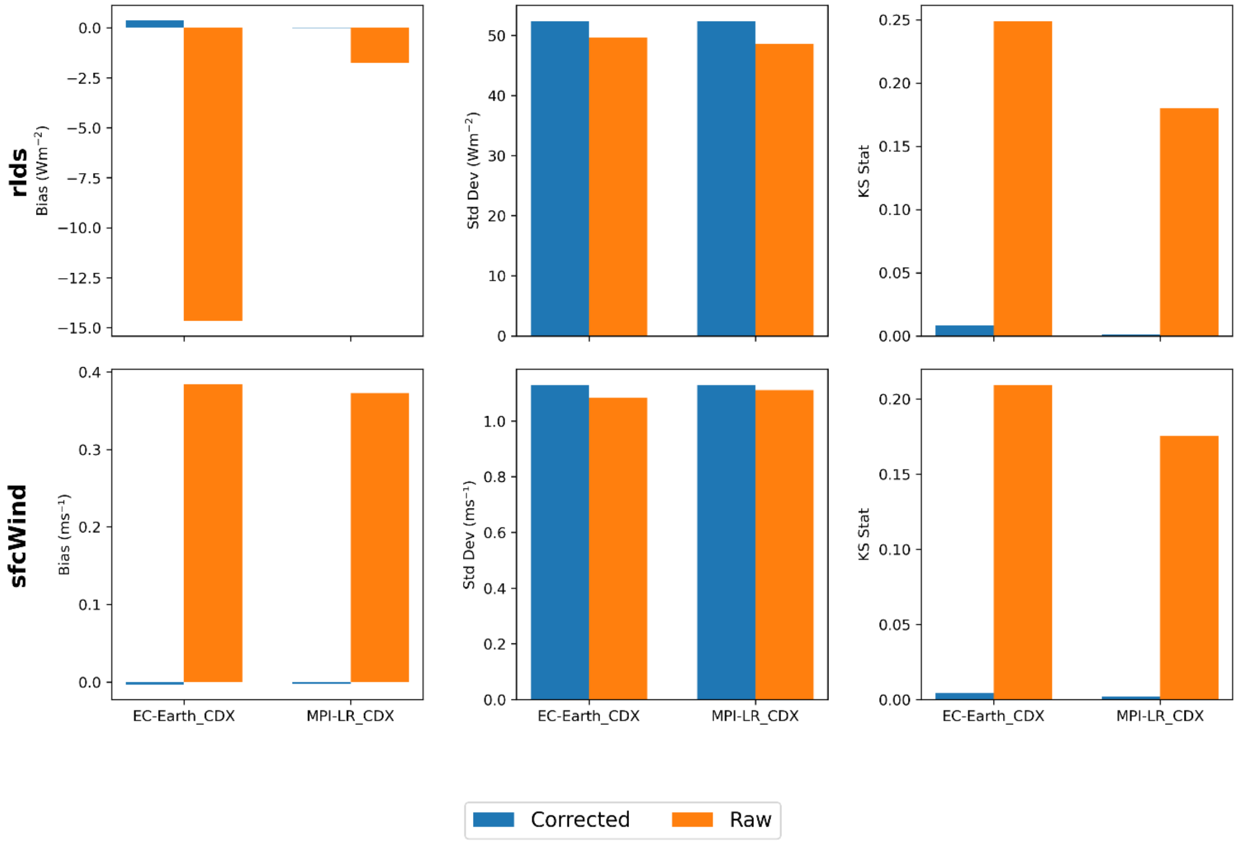

Figure 7. Same as Figure 6, but for rlds and sfcWind.

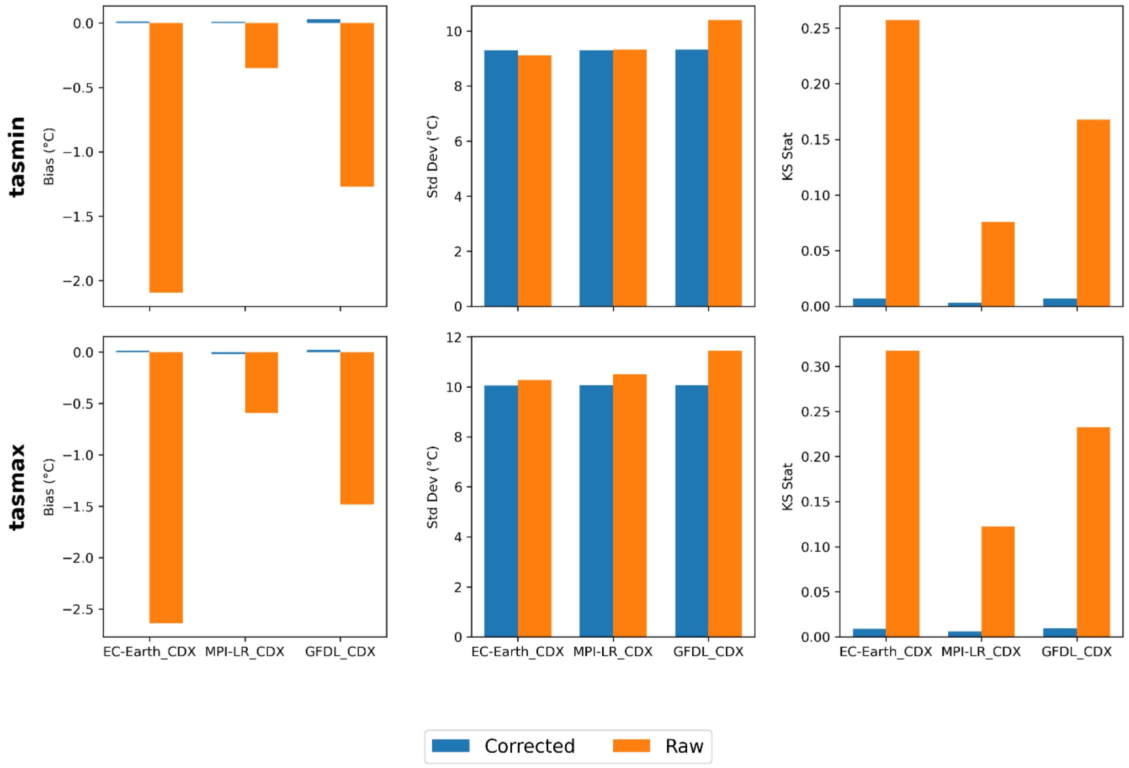

Figure 8. Same as Figure 6, but for tasmin and tasmax.

References

1 Taylor, K. E. Summarizing multiple aspects of model performance in a single diagram. *Journal of geophysical research: atmospheres* **106**, 7183-7192 (2001).

2 Yakubu, F., Böhner, Jürgen, Schickhoff, Udo, Scholten, Thomas, & Hasson, Shabeh Ul. Global Bias-Corrected CORDEX Datasets at Half Degree Resolution (Version 2025_fv0.01) [Data set]. (2025). https://doi.org/10.25592/uhhfdm.17560
